# Therapeutic Effects of Zoledronic Acid-Loaded Hyaluronic Acid/Polyethylene Glycol/Nano-Hydroxyapatite Nanoparticles on Osteosarcoma

**DOI:** 10.3389/fbioe.2022.897641

**Published:** 2022-05-26

**Authors:** Yan Xu, Jingqi Qi, Wei Sun, Wu Zhong, Hongwei Wu

**Affiliations:** ^1^ Hunan Cancer Hospital and The Affiliated Cancer Hospital of Xiangya School of Medicine, Central South University, Changsha, China; ^2^ Zhejiang University-University of Edinburgh Institute, Haining, China

**Keywords:** nanoparticle, osteosarcoma, zoledronic acid, tumor therapy, targeted therapy

## Abstract

Zoledronic acid (ZOL) has been approved as the only bisphosphonate for the prevention and treatment of metastatic bone diseases with acceptable safety and tolerability. However, systemic or direct injection of ZOL often causes severe side effects, which limits its clinical application. Here, an innovative nano-drug delivery system, ZOL-loaded hyaluronic acid/polyethylene glycol/nano-hydroxyapatite nanoparticles (HA-PEG-nHA-ZOL NPs), has been found to effectively inhibit the proliferation of three types of human osteosarcoma cell lines (143b, HOS, and MG63) at 1–10 μmol/L, while with low cell cytotoxicity on normal cells. The NPs significantly enhanced the apoptosis-related protein expression and tumor cell apoptosis rate. The NPs could also inhibit the proliferation of osteosarcoma cells by blocking the S phase of the cell cycle. In the orthotopic osteosarcoma nude mice model, local injection of the HA-PEG-nHA-ZOL NPs stimulated tumor necrosis, apoptosis, and granulocyte infiltration in the blood vessels. Altogether, the ZOL nano-delivery system possesses great potential for local treatment to prevent local tumor recurrence and can be applied in clinical osteosarcoma therapy.

## Highlights

1) Newly synthesized zoledronic acid-loaded organic–inorganic hybrid nanoparticles have specific inhibiting effects on different types of osteosarcoma cell proliferation, while their effect on normal cells is insignificant.
2) Local injection of the nanoparticles *in vivo* significantly increased intratumoral vascular inflammation, which mediated the tumor tissue necrosis and apoptosis.
3) Our findings provide a new approach for local therapy to treat osteosarcoma.

## 1 Introduction

Osteosarcoma is the most prevalent primary malignant tumor of the bone in children and young adults with an estimated incidence of 3 cases/million population per year and a peak of incidence at 11–15 years (Mirabello et al., 2009; Travis et al., 2015). Osteosarcoma commonly happens in the distal femur, proximal tibia, and humerus, which hinders patients’ movement seriously (Meazza and Scanagatta, 2016; Letaief et al., 2020). Lung metastasis arisen from osteosarcoma, leading to metastatic and recurrent diseases, attributes to the treatment failures and high mortality of osteosarcoma (Yu et al., 2017). Long-term survival at 5 years in patients with local osteosarcoma tumors is approximately 60%, and it significantly reduces to 20% if pulmonary metastasis occurs at the time of diagnosis (Oertel et al., 2010). Thus, novel efficient therapies against osteosarcoma are in urgent need.

Clinically, surgery is an integral part of treatment for patients with localized osteosarcoma and patients with metastatic or recurrent osteosarcoma. Advances in imaging, new biomaterials, and prostheses have provided surgeons with more accurate preoperative planning and a wider range of surgical options, encouraging the development of surgical techniques and less aggressive interventions (Anderson, 2016). After excision or curetting of the lesion, bone cement is used to supplement the plate after excision or curetting of the lesion (Katagiri et al., 1997). As a common bone cement, nano-hydroxyapatite (nHA) possesses a similar molecular structure and calcium/phosphorus ratio to the natural bones of vertebrates. Additionally, the incorporation of nHA can promote bone regeneration through the osteoinductive effects on bone marrow mesenchymal stem cells (Liu et al., 2015). Thanks to its good bone conductivity and non-toxicity, nHA has been widely used in bone tissue repair (Hernigou et al., 1989; MacMillan et al., 2014). However, the residual osteosarcoma cells after resection surgery may lead to continued bone destruction, new lesions in surrounding tissues, and even osteosarcoma recurrence. In order to reduce the risk of local recurrence, the addition of chemotherapeutic agents to bone cement as an adjunct therapy for bone metastases has been recommended. For instance, methotrexate implanted in nHA cement has shown local and general therapeutic effects on rats with induced osteosarcoma (Hernigou et al., 1989). Altogether, nHA can function as a drug-carrying scaffold to be applied in the adjunct therapy against osteosarcoma.

Bisphosphonates, inorganic pyrophosphate analogs, possess inhibitory effects on tumor-induced osteolysis and bone metastasis (Nadar et al., 2017). Zoledronic acid (ZOL), a third-generation bisphosphonate, exhibits prominent effects on tumor cell apoptosis promotion, angiogenesis inhibition, and metastasis inhibition (Chang et al., 2012; Ohba et al., 2014a; Wang et al., 2020). ZOL can induce the S phase arrest and apoptosis accompanied by DNA damage and activation of the ataxia-telangiectasia-mutated/checkpoint kinase 1/cell division cycle 25 pathway to inhibit the growth of osteosarcoma cells (Iguchi et al., 2007). Additionally, ZOL has been reported to suppress the platelet-derived growth factor-BB secreted by preosteoclasts, resulting in angiogenesis and osteogenesis inhibition that can block tumor metastasis (Gao et al., 2017). However, ZOL has severe adverse effects including osteoporosis-related fractures, renal impairment, and serious atrial fibrillation that impose restrictions in clinical application (Dhillon, 2016; Reyes et al., 2016). ZOL possesses promising potential in anti-osteoblastoma, but the dosage, application protocol, and delivery system need to be strictly controlled in future clinical use.

Non-specific targeting therapies of tumor including surgery, chemotherapy, radiotherapy, and hormone therapy show poor therapeutic effects with high dosage-induced side effects. Nanoparticles (NPs), a new recognition tool, can deliver the drug to target tumor cells and minimize the chance of drug leakage to normal cells (Bahrami et al., 2017). NP binding with tumor-specific biomarker ligands is an effective method to control antitumor drugs to reach the tumor area and achieve maximum efficacy treatment. Osteosarcoma cells highly express the cluster of differentiation 44 (CD44), which is a receptor for hyaluronic acid (HA), and the HA-binding participates in various tumor activities, including tumor progression, metastasis, and drug resistance (Xiao et al., 2018). Nevertheless, intravenously administered particles are usually cleared by the reticuloendothelial system within minutes of administration and finally accumulate in the liver and spleen (Gref et al., 1995). In order to prevent the opsonization of macrophages, polyethylene glycol (PEG) can be used to form a hydrated film on the surface of NPs to reduce the clearance in plasma and improve drug utilization (Gref et al., 1995). Moreover, the relatively high biocompatibility and hydrophilicity of PEG make it more favorable in the NP drug delivery system (Arcaute et al., 2006). In normal tissues, the microvascular endothelial space is dense with intact structures, and thus, macromolecules and lipid particles cannot easily penetrate the vascular wall. According to the enhanced permeability and retention (EPR) effect, the solid tumor tissues possess abundant blood vessels with a broad inner diameter, poor structural integrity, and lack of lymphatic reflux, resulting in high permeability and retention of macromolecules and lipid particles (Wu, 2021). Following this, NPs with a size of 50–500 nm can penetrate the capillary wall of bone tumors and realize their enrichment in tumor tissue, indicating a passive targeting behavior (Blanco et al., 2015). As the size increases beyond 150 nm, more NPs are entrapped within the liver and spleen, and small-sized NPs (less than 5 nm) are commonly filtered out by the kidneys (Longmire et al., 2008). In this study, we designed nearly 100-nm inorganic NPs modified with HA and PEG on nHA, which can load ZOL to target osteosarcoma cells. Based on the targeting ability, the HA-PEG-nHA-ZOL NPs can achieve the timing, positioning, and quantitative release of drugs, bringing about positive therapeutic effects.

## 2 Materials and Methods

### 2.1 Materials

Low-molecular weight hyaluronic acid (HA) was purchased from Shandong Freda Biotechnology Co., Ltd. Dimethyl sulfoxide and deuterium oxide were purchased from Sinopharm Chemical Reagent Co., Ltd. Dialysis bags (7000 kda and 8000–14000 kda) were obtained from Shanghai Yuanye Bio-Technology Co., Ltd. Polyethylene glycol (PEG), tetrabutylammonium hydroxide (TBA, 40% in H2O), and N, N′-dicyclohexylcarbodiimide (DCC) were purchased from Aladdin Reagent (Shanghai). Dulbecco’s modified Eagle medium/high modified (DMEM-H), phosphate-buffered saline (PBS), antibiotic–antimycotic, and fetal bovine serum (10099-141) were purchased from Gibco. Human osteosarcoma cell lines 143b, MG63, and HOS were obtained from Shanghai Genechem Co., Ltd. Human skin fibroblasts (HSFs) were purchased from the ATCC (Jennio-bio, Guangzhou, China). Rat adipose-derived stem cells (ASCs) and bone marrow mesenchymal stem cells (BMSCs) were isolated from healthy rat adipose tissues and femur marrow, respectively. The LIVE/DEAD Cell Imaging Kit (488/570) was purchased from Thermo Fisher.

### 2.2 Methods

#### 2.2.1 Synthesis of Hyaluronic Acid–Tetrabutylammonium Hydroxide

First, 1 g HA was dissolved in 20 ml of deionized water in a small beaker. Then, 1 ml TBA was added to the HA solution, and the solution was stirred at room temperature for half an hour. The solution was then frozen at −20°C and freeze-dried in a vacuum lyophilizer to obtain the HA-TBA polymer.

#### 2.2.2 Synthesis of Hyaluronic Acid–Polyethylene Glycol Nano-Hydroxyapatite

Briefly, 1 g HA-TBA polymer, 0.5 g PEG, and 0.5 g nHA were added into a small flask, and 30 ml DMSO liquid was added to dissolve these solids. The resulting solution was ultrasonically treated with an ultrasonic disperser in an ice bath for 60 min, and then 0.5 g DCC was added. The container was transferred to an oil bath at 50°C and stirred for 2 days. The reaction liquid was added into a 50-ml centrifuge tube and centrifuged at 5,000 rpm for 20 min. The supernatant was removed, and the particles were dispersed in 10 ml DMSO with an ultrasonic disperser. The suspension was then added to a dialysis bag (8000–14000 kda) and the deionized water was replaced every half hour. After 12 h, liquid in the dialysis bag was removed and lyophilized to obtain the HA-PEG-nHA material.

#### 2.2.3 Preparation of Zoledronic Acid-Loaded Nanoparticles

At first, 10 mg HA-PEG-nHA material was weighed and added to 10 ml PBS for 1-min ultrasound adoption to obtain HA-PEG-nHA NPs. Based on the adsorption method, 10 ml deionized water was added to 0.5 g HA-PEG-nHA material and 0.2 g ZOL. After 1-min ultrasonic dispersion of the solution, stirring was performed for 4 h. Then, the solution was centrifuged at 5,000 rpm for 20 min, and the precipitate was lyophilized to obtain HA-PEG-nHA-ZOL NP powder (Xu et al., 2021).

#### 2.2.4 Nuclear Magnetic Resonance

Briefly, 10 mg HA-PEG-nHA-ZOL NPs and 10 mg ZOL were dispersed in 1 ml deuterated heavy water, and the drug loading of HA-PEG-nHA-ZOL NPs was calculated by using a nuclear magnetic resonance proton spectrometer. The calculation formula is as follows:

$${\text{A}}_{\text{i}}{=\text{A}}_{\text{δ}7.26}+{\text{A}}_{\text{δ}7.41},$$

$$\mathrm{DS}=\frac{{\text{A}}_{\text{Zol}}}{{\text{A}}_{\text{NPs}}}\times 100\text{\%,}$$

where i = ZOL or HA-PEG-nHA-ZOL NPs, and ${\text{A}}_{\text{δ}7.26}{\text{ and A}}_{\text{δ}7.41}$ are both characteristic hydrogen peaks on the imidazole group. The NMR was measured three times, and the average was calculated.

#### 2.2.5 Transmission Electron Microscopy

HA-PEG-nHA-ZOL NPs containing 2% phosphoric acid were dropped onto a copper wire coated with a carbon supporting film. After drying, the morphology was observed under a transmission electron microscope.

#### 2.2.6 Cell Culture

Human osteosarcoma cells (143b, MG63, and HOS) and human skin fibroblasts (HSFs) were cultured in a carbon dioxide incubator. The culture medium was DMEM-H containing 1% antibiotic–antifungal and 10% FBS. When the cells in the culture flask reached 90%, the passage was carried out. The cells after the seventh generation were used for cell testing.

#### 2.2.7 Cytotoxicity *In Vitro*

Cell viability was detected with a CCK-8 kit. HSFs, ASCs, BMSCs, 143b, HOS, and MG63 cells were inoculated into 96-well plates (5,000 cells each well), with 6 wells in each group. After 1 day of culture, NPs with various concentrations were added and then cultured for another 3 days, and then 100 μL CCK-8 was added for 3 h. The absorbance at 450 nm was measured with a microplate card reader, and the cell viability was calculated as follows:

$$\text{viability rate }\left(\text{\%}\right)=\frac{\text{OD}\left(\text{Experimental Group}\right)-\text{OD}\left(\text{Blank group}\right)}{\text{OD}\left(\text{Control group}\right)-\text{OD}\left(\text{Blank group}\right)}\times 100\text{\%}\text{,}$$

#### 2.2.8 Nanoparticle *In Vivo* Injection

This study was performed in accordance with the prevailing regulations on the protection of animals used in experiments. The study was approved by the Hunan Cancer Hospital Ethnic Committee on Animal Use (No. 2018-11). The orthotopic osteosarcoma model of nude mice was established according to the previous method (Wu et al., 2021a). After the tumor grew to about 1.5 cm^3^, 50 μL (200 ug) NPs, or saline was locally injected into the tumor, respectively. Five mice were used in each group. Five days later, specimens were obtained for subsequent histology analysis.

#### 2.2.9 Histology Analysis

Five days later, the mice were killed, and specimens including tumor, lungs, liver, spleen, and kidneys were collected. The specimens were fixed with 4% paraformaldehyde overnight. Then, the specimens were dehydrated and embedded in paraffin. They were sectioned into 5-μm slices and stained with hematoxylin and eosin (H&E). The stained sections were imaged under an inverted phase-contrast microscope (Olympus CX23, Japan).

#### 2.2.10 Immunohistochemistry Staining

Tumor specimens were further immunostained with TUNEL (G1507, Servicebio), Ki67 (G111141, Servicebio), and caspase 3 (GB11532, Servicebio) to detect the apoptosis state of the tumor. Dewaxed and rehydrated paraffin sections were first treated with 3% hydrogen peroxide in methanol solution for 15 min. After PBS flush, the sections were blocked with 10% secondary antibody serum for 30 min. Then, diluted primary antibodies (1:100) were added, and the solution was incubated overnight at 4°C. After PBS flush, a secondary antibody was added, and the solution was incubated for 30 min at room temperature. Accordingly, DAB coloration was performed, and hematoxylin was applied to restain the nuclei.

### 2.3 Statistical Analysis

Statistical analysis was performed with Origin 2017 and GraphPad Prism 7. Most of the results were expressed as mean with a 95% confidence interval.

## 3 Results

### 3.1 Fabrication and Characterization of HA-PEG-nHA-ZOL Nanoparticles

**FIGURE 1:**
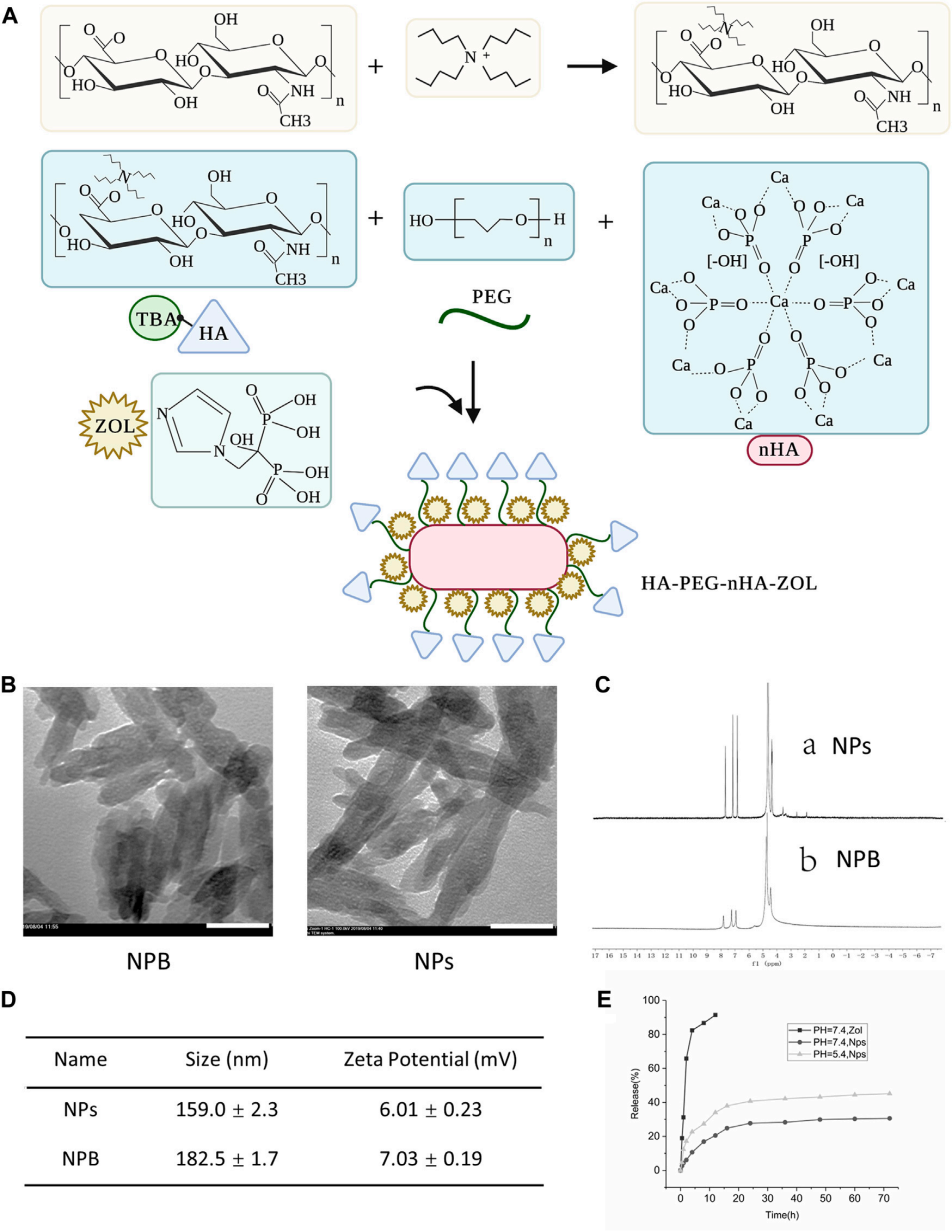
Characterization of blank nanoparticles (NPB) and zoledronic acid-loaded nanoparticles (NPs). **(A)** Schematic diagram of the construction of nanoparticles. **(B)** TEM images of blank nanoparticles and drug-loaded nanoparticles. Scale bar = 50 nm. **(C)** Magnetic resonance images of drug-loaded nanoparticles and blank nanoparticles. **(D)** Average size and zeta potential of the nanoparticles. **(E)** Drug release curve of the nanoparticles.

To construct the HA-PEG-nHA-ZOL NPs, several steps were taken, as shown in Figure 1. First, the mixture of TBA and HA was lyophilized to obtain the HA-TBA polymer. The HA-TBA polymer was then mixed with PEG and nHA and subjected to ultrasonic treatment, oil bath, centrifugation, and lyophilization. The product, HA-PEG-nHA material, was adopted through ultrasound to obtain HA-PEG-nHA NPs. After ZOL was absorbed by HA-PEG-nHA NPs, HA-PEG-nHA-ZOL NP powder was obtained by ultrasonic dispersion, centrifugation, and freeze-drying. Accordingly, the mixed NPs were composed of an nHA core that recruited hydrophilic ZOL molecules and encapsulated them in a PEG-HA polymer shell. Transmission electron microscopy images showed that the blank (NPB) and ZOL-loaded NPs had a rod-like geometric profile with a length of about 150 nm and a cross-sectional diameter of about 40 nm (Figure 1B). The drug loading process and efficacy were determined by nuclear magnetic resonance (NMR) (Figure 1C). The peak at 4.65 ppm refers to the methylene peak on ZOL, 3–4 ppm is the methyl peak on HA and PEG, 1.9–2.6 ppm stands for the hydroxyl peak on HA, and 7–8 ppm is the characteristic peak of hydrogen on the imidazole ring. The average size and zeta potential of the nanoparticles are shown in Figure 1D. The drug release curve is shown in Figure 1E, which had been reported in our previous study (Xu et al., 2021).

### 2.3.2 The Cytotoxicity Effect of HA-PEG-nHA-ZOL NP on Osteosarcoma Cells

**FIGURE 2:**
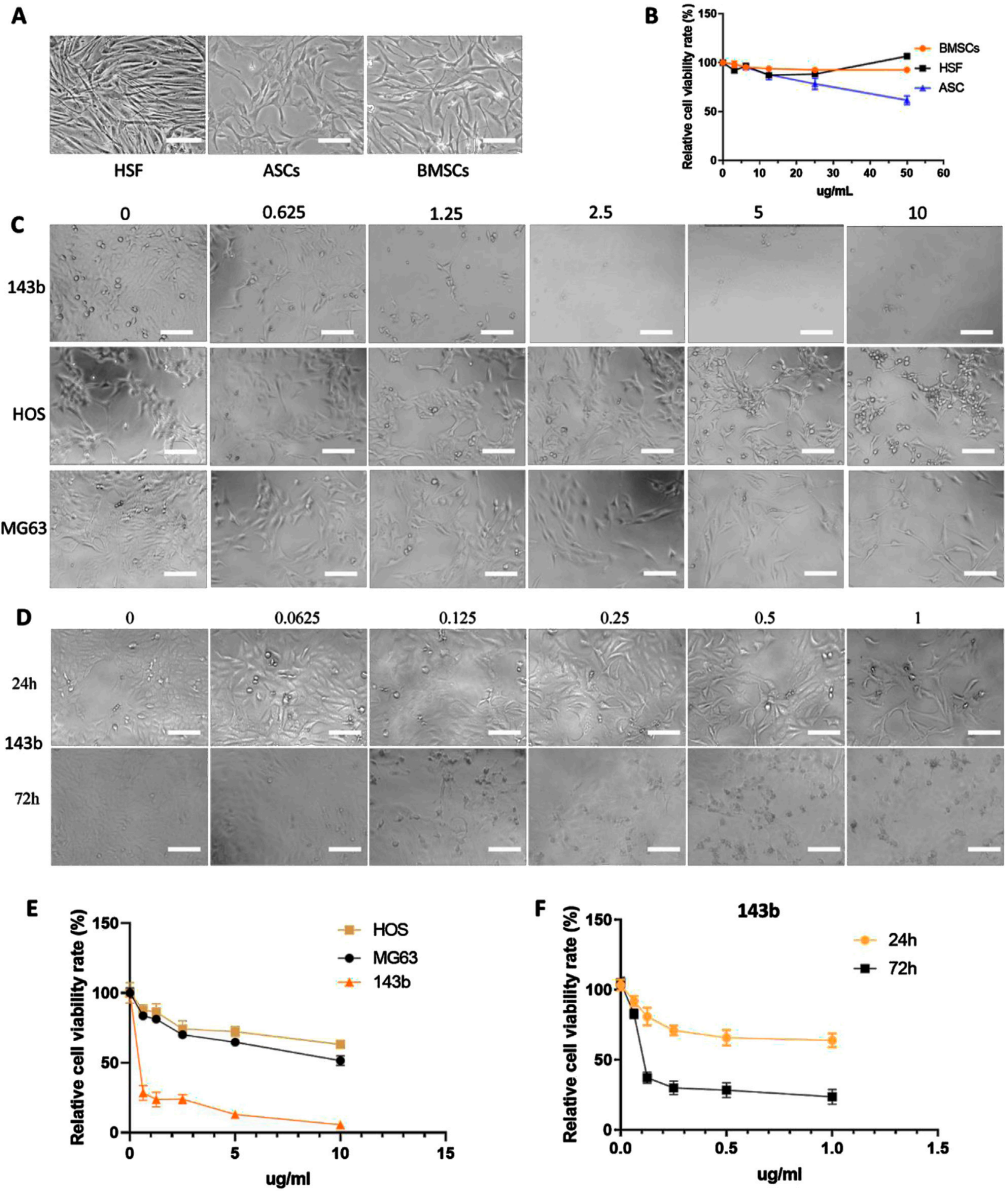
Effect of nanoparticles on three types of normal cells and three osteosarcoma cell lines. **(A)** Photoscopic images of three normal cell types (HSFs, ASCs, and BMSCs) treated with 0 -50 μg/mL concentrations of nanoparticles. **(B)** Relative cell viability of three normal cell lines under 0 -50 μg/mL concentrations of nanoparticles. **(C, E)** Photoscopic images and relative cell viability of three kinds of osteosarcoma cells (143b, HOS, and MG63) treated with 0 -10 μg/mL concentrations of nanoparticles. **(D, F)** Photoscopic images and relative cell viability of 143b cells treated with 0 -1 μg/mL concentrations of nanoparticles. Scale bar = 100 um.

To detect the cytotoxicity effect of HA-PEG-nHA-ZOL NPs on normal cells, three normal cell types (HSFs, ASCs, and BMSCs) were selected and treated with high concentrations (0–50 μg/ml) of NPs (Figure 2A). The NPs did not show significant effects on the cell viability of BMSCs or HSF, although ASCs reduced slightly after the NP treatment of 10 μg/ml (Figure 2B). Since the effects of high concentrations (10–50 μg/ml) of NPs on normal cells are insignificant, the effects of low concentrations (0–10 μg/ml) of NPs on normal cells can be ignored. Then, 0–10 μg/ml concentrations of synthesized NPs were placed into three common osteosarcoma cell lines (143b, HOS, and MG63)(Figure 2C). As a result, the amounts of the three types of tumor cells decreased significantly with the increase in the NP concentration, while the 143b cell line possessed dominant reduction (Figure 2E). To further detect the appropriate concentration of HA-PEG-nHA-ZOL NPs, concentration gradients from 0 μg/ml to 1 μg/ml were applied in the 143b cell culture separately (Figure 2D). When the HA-PEG-nHA-ZOL NP concentration was increased from 0 to 0.125 μg/ml, the cell number decreased rapidly, and after 0.125 μg/ml, the cell number remained constant with the increase in concentration. Compared with the 24-hour NP treatment, the survival cell number dropped dramatically under the 72-hour NP exposure, indicating that the NPs inhibited cell survival significantly (Figure 2F). These results indicated that the HA-PEG-nHA-ZOL NPs targeted a series of osteosarcoma cells, and the HOS and MG63 cell lines exhibited less sensitivity to the NPs than the 143b cell line.

### 3.3 HA-PEG-nHA-ZOL NPs Inhibit the Apoptosis of Osteosarcoma Cells

**FIGURE 3:**
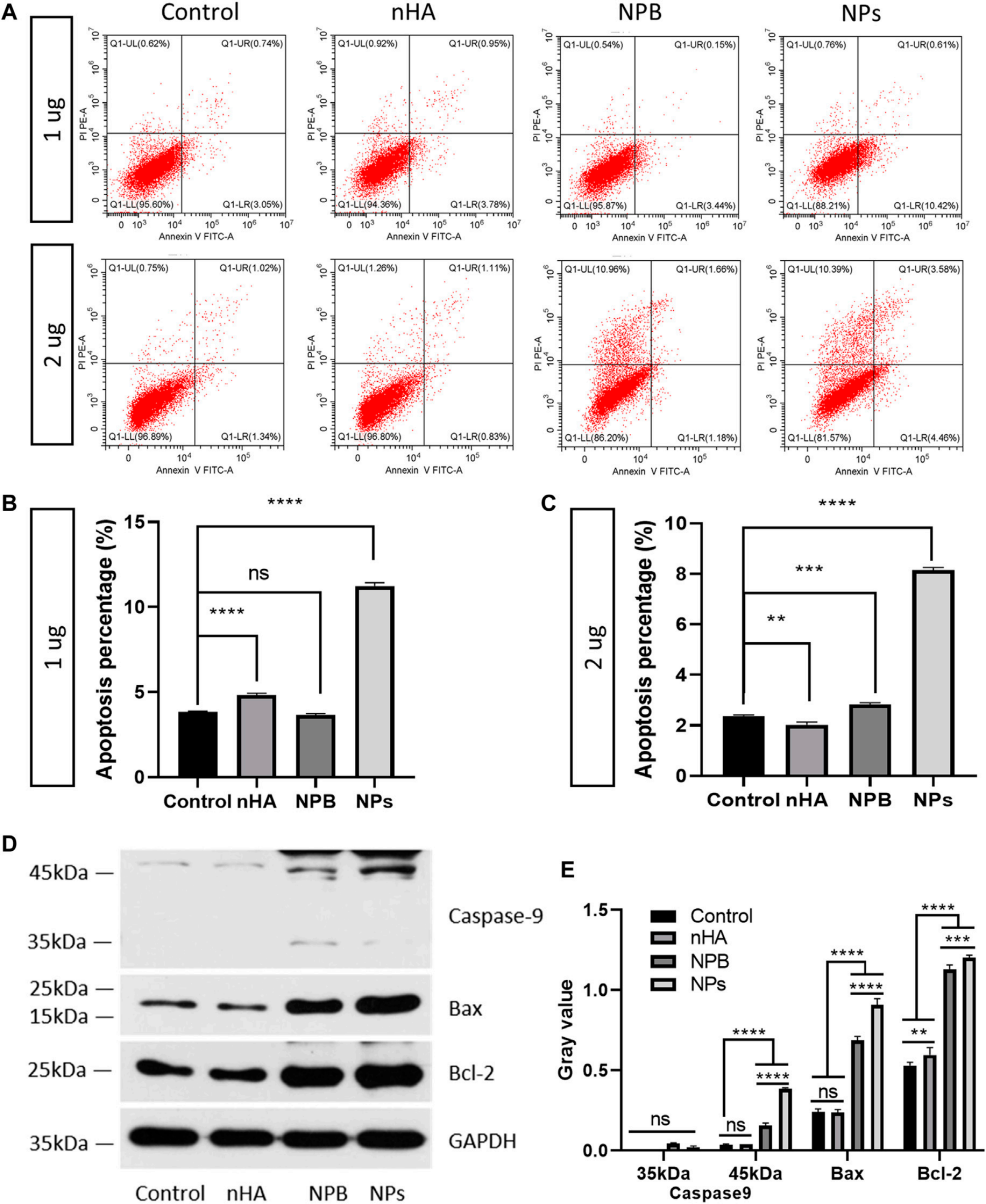
Nanoparticles promote the apoptosis of osteosarcoma cells. **(A)** Flow cytometry results of 143b cells treated with 1 and 2 μg nanoparticles. **(B,C)** Apoptosis percentage of 143b cells treated with 1 and 2 μg nanoparticles. **(D,E)** Western blot band and gray value of apoptosis related proteins. ns: non-significance, **p < 0.01, ***p < 0.001, ****p < 0.0001.

To detect the effect of HA-PEG-nHA-ZOL NPs on osteosarcoma cell apoptosis, nHA, NPB, and cell medium without a reagent was selected as control groups. Two concentrations (1 and 2 μg) of HA-PEG-nHA-ZOL NPs, nHA, and NPB were added to the 143b cell medium, respectively. Using Annexin V-FITC apoptosis staining, the apoptosis percentages of the NP groups of both 1 and 2 μg were significantly higher than those of the other three groups (Figures 3A–C). As mentioned before, the 143b cell lines possessed high sensitivity and low tolerance to the ZOL stimulation; thus, most of the cells were apoptotic under high dosage of the drug that decreased the apoptosis percentage. Then, sodium dodecyl sulfate–polyacrylamide gel electrophoresis was performed to detect apoptosis-associated proteins (Figure 3D). After quantitative analysis, caspase 9, a member of the caspase family of cysteine proteases involved in the processing of cell apoptosis, was highly expressed in the NP groups (Figure 3E). Also, the NP groups possessed the highest expression level of Bcl-2-associated X protein (BAX), indicating that the NP induced more activated gateways to cell death via the mitochondria. As for the B-cell lymphoma protein 2 alpha (Bcl-2), it is an antiapoptotic protein located primarily in the outer mitochondrial membrane that blocks the apoptotic death of cells such as lymphocytes. Although both NPB and NP groups showed higher levels of Bcl-2 than the nHA and control groups, the NP groups showed higher gray values than that of the NPB groups. Taken together, the HA-PEG-nHA-ZOL NPs have been demonstrated to inhibit the apoptosis of osteosarcoma cells.

### 3.4 HA-PEG-nHA-ZOL NPs Affect the Osteosarcoma Cell Cycle

**FIGURE 4:**
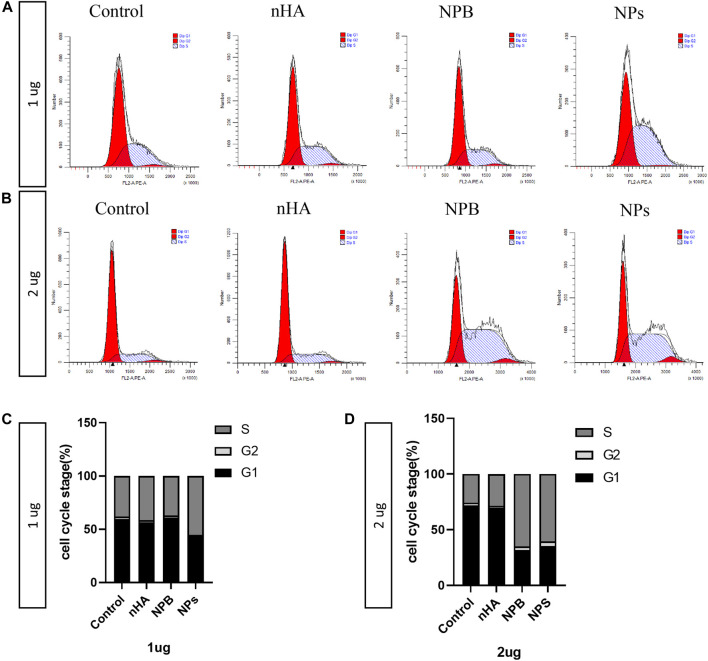
Nanoparticles affect the cell cycle of osteosarcoma. **(A)** Cell cycle results of 143b cells treated with 1 μg/ml of nanoparticles. **(B)** Cell cycle results of 143b cells treated with 2 μg/ml of nanoparticles. **(C)** Distribution of the cell cycle stages of 143b cells treated with 1 μg/ml nanoparticles. **(D)** Distribution of cell cycle stages of 143b cells treated with 2 μg/ml nanoparticles.

**FIGURE 5:**
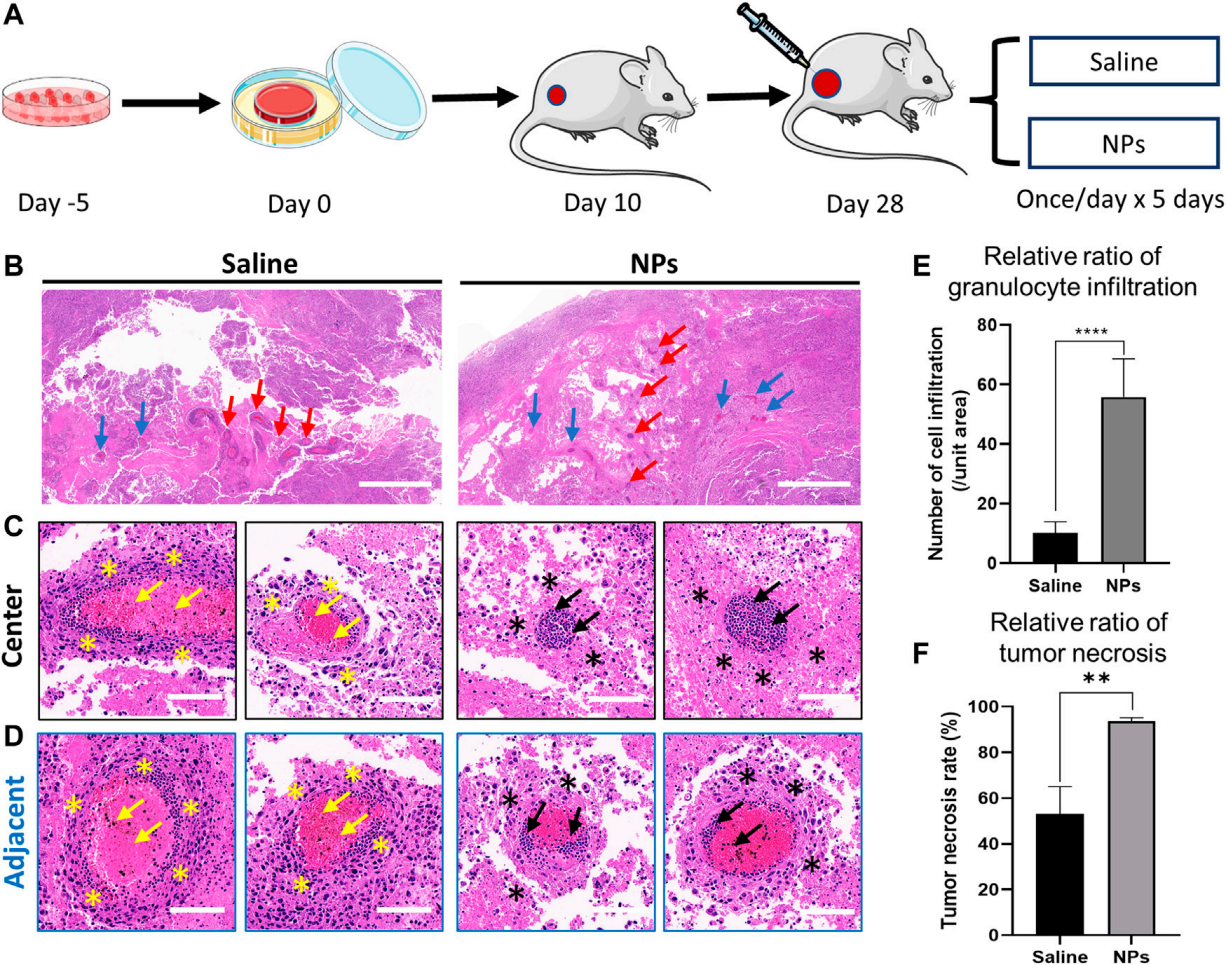
Nanoparticles induce osteosarcoma cell necrosis and vascular inflammation in the tumor. **(A)** Schematic diagram of nanoparticle and saline injections in an orthotopic osteosarcoma model. **(B)** Representative images of H&E staining after intra-tumor injection in the saline and nanoparticle groups. Red arrows indicate the central area of the injection. Blue arrows indicate the adjacent area of the injection. Scale bar = 1 mm. **(C, D)** H&E staining of granulocyte infiltration around the vascular space in the central and adjacent areas of the puncture site. Yellow and black arrows indicate the granulocyte infiltration in the vessel. Yellow and black stars indicate the tumor necrosis around the vessel. **(E)** Comparison of granulocyte infiltration between saline and nanoparticle groups (****p < 0.0001). **(F)** Comparison of tumor necrosis around the vessel between saline and nanoparticle groups (**p < 0.01). Scale bar = 100 μm.

Since HA-PEG-nHA-ZOL NPs have been shown to induce apoptosis of osteosarcoma cells, the underlying mechanism of the cell cycle process requires to be found out. Flow cytometry was performed to study different stages of the cell cycle from subpopulations of cells to detailed cell kinetic information. In the case of 1 μg, the S stage of the NP groups was longer than the control, nHA, and NPB groups (Figures 4A,C). As for 2 μg of dosage, both NPB and NP groups showed an extended S stage than the control and nHA groups (Figures 4B, 5D). The S phase of a cell cycle occurs during the interphase and is responsible for the synthesis of DNA. The NP-induced blockage of the S phase may inhibit the proliferation of osteosarcoma cells.

### 3.5 HA-PEG-nHA-ZOL NPs Inhibit the Proliferation of Osteosarcoma Cells *In Vivo*

Briefly, 50 μl of saline and NPs (200 μg) were injected into the osteosarcoma nude mice models implanted with tumor cell sheets once a day continuously for 5 days (Figure 5A). Hematoxylin and eosin (H&E) staining was performed to evaluate the *in vivo* toxicity. In the NP groups, inflammatory cells were largely distributed along the injection central area, and there were fewer inflammatory cells in the margin area (Figures 5B–D). The tumor necrosis rate around the vessels in the NP groups was much higher than that in the saline group (Figure 5F, *p* < 0.01). The results suggested that the nanoparticles induced high granulocyte infiltration in the vessel and tumor necrosis in the tumor tissue. After the saline injection, the distribution of inflammatory cells in the center and margin was dense and scattered, and the number of inflammatory cells was less than that in the NP groups. The quantitative granulocyte infiltration result shows that NPs caused greater granulocyte infiltration both in central and adjacent areas (Figures 5E,F). Following these, the NP injection mainly induced violent local inflammation, which may be evoked by the necrosis of tumor cells (Proskuryakov and Gabai, 2010), causing tumor repression.

### 3.6 HA-PEG-nHA-ZOL NPs Show Local Antitumor Effects *In Vivo*

**FIGURE 6:**
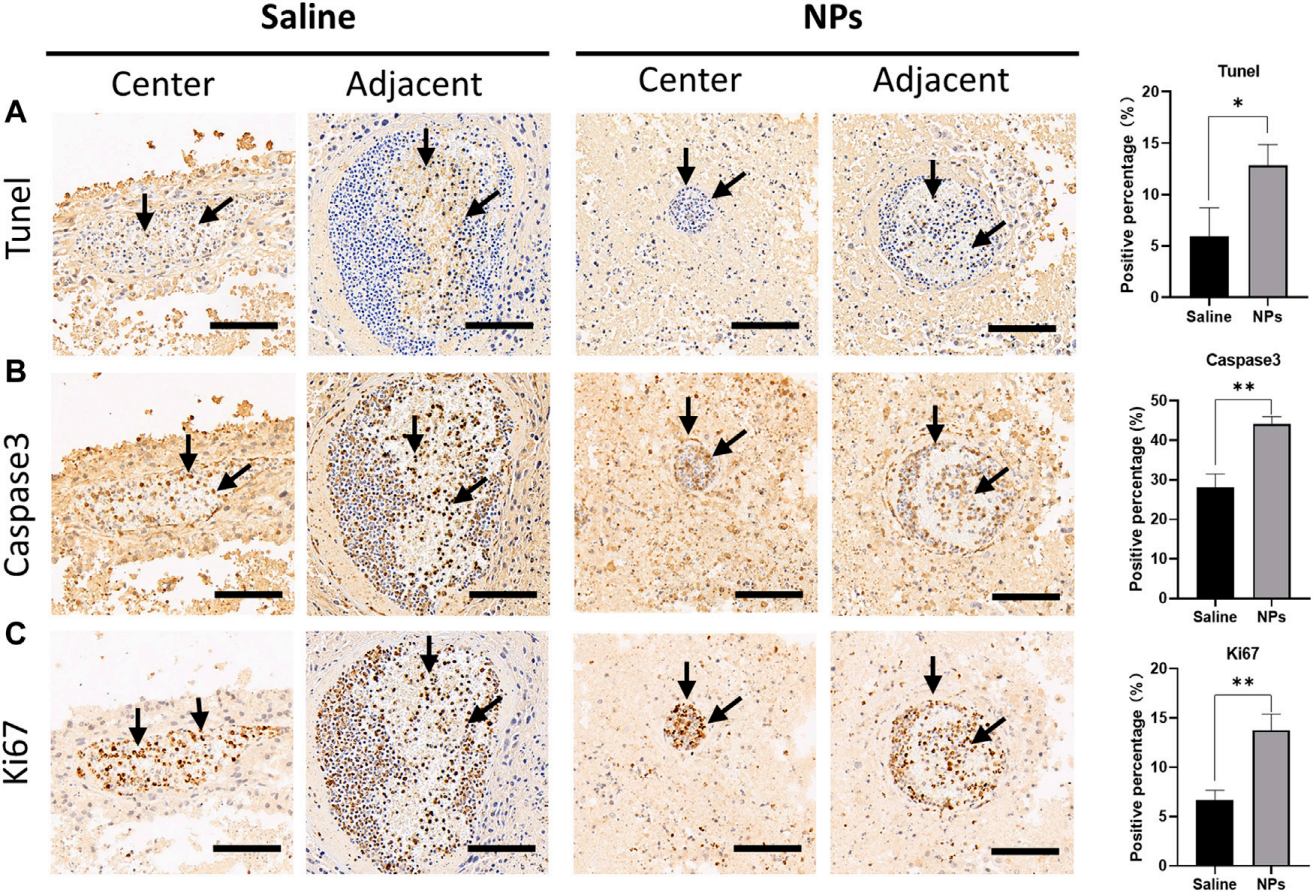
Immunohistochemical staining of tumor apoptosis indicators after injection of nanoparticles in the tumor. **(A)** Microscopic photos of TUNEL staining in the central and adjacent areas of the puncture site in saline and nanoparticle injection groups and comparison of the positive percentage between the two groups (*p < 0.05). **(B)** Microscopic photos of caspase 3 staining in the central and adjacent areas of the puncture site in each group and comparison of the positive percentage between the two groups (**p < 0.01). **(C)** Microscopic photos of Ki67 staining in the central and adjacent areas of the puncture site in each group and comparison of the positive percentage between the two groups (**p < 0.01). Scale bar = 100 um.

Immunohistochemical staining was conducted, and the proportions of the malignant cells staining positive for the TUNEL, nuclear antigen Ki67, and caspase 3 were evaluated using light microscopes. TUNEL and caspase 3 staining were performed to detect the cell apoptosis characterized by nuclear DNA fragmentation to nucleases. Ki67, a nuclear antigen presenting throughout the active cell cycle, can indicate an increase in the cell turnover. NP injections have been found with higher levels of TUNEL and caspase 3 than the saline injections (Figures 6A,B), suggesting that NPs could induce tumor cell apoptosis and inhibit the division of tumor cells. Ki67 immunohistochemical staining demonstrates that the expression of the cell proliferation index of the NP groups was mainly concentrated in blood vessels, while Ki67 expression was distributed randomly in saline groups (Figure 6C). In addition, the NP groups possessed higher levels of Ki67 than the saline group, indicating that NP injections caused increased aggregation of inflammatory cells in blood vessels. As mentioned previously, NP-induced inflammation could promote the apoptosis and necrosis of tumor cells.

### 3.7 HA-PEG-nHA-ZOL NPs Have No Impact on the Lung, Liver, Spleen, and Kidney

**FIGURE 7:**
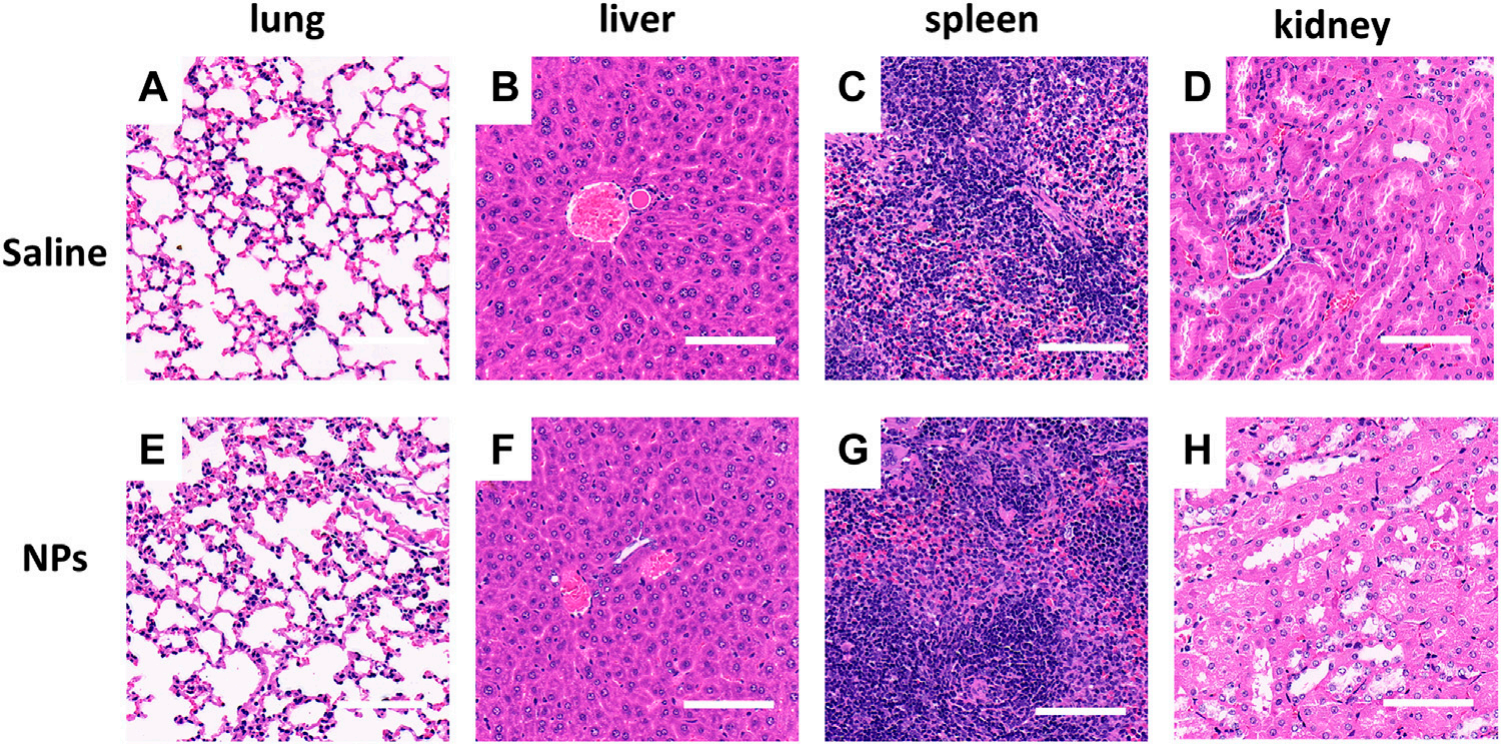
Nanoparticles showed no organ toxicity after local intra-tumor injection. **(A–D)** Microscopic photos of H&E staining of organs (lung, liver, spleen, and kidney) in the saline injection group. **(E–H)** Microscopic photos of H&E staining of organs (lung, liver, spleen, and kidney) in the nanoparticle injection group. Scale bar = 200 μm.

After 5 days of NP injection, H&E staining was performed to observe the impact of HA-PEG-nHA-ZOL NPs on the lung, liver, spleen, and kidney tissues. Compared with the saline group (Figures 7A–D), there was no significant difference between the NP groups and the saline group in terms of the degree of edema, granulocyte infiltration, and degeneration and necrosis in the tissue (Figures 7E–H), indicating that the local treatment of HA-PEG-nHA-ZOL NPs did not cause systemic adverse effects.

## 4 Discussion

Osteosarcoma is considered the most common primary bone malignancy. Currently, the main clinical treatment mode is “preoperative adjuvant chemotherapy + surgical resection + postoperative adjuvant chemotherapy” (Wu et al., 2021b). However, traditional chemotherapeutic drugs have various side effects and poor targeting ability, which significantly reduce the quality of patients’ life (Burns and Wilding, 2020). Osteosarcoma-mediated osteolysis has been considered the main cause of high mortality (Ohba et al., 2014b). Due to the good anti-osteolysis activity, anti-proliferation, antiangiogenic, and immunomodulatory effects against tumor, ZOL, a third generation of bisphosphonate, has been used for bone metastasis of various malignant tumors including breast cancer and prostate cancer (Wu et al., 2021b) (Ouyang et al., 2018). However, the inhibitory effect of ZOL on tumor growth and lung metastasis of osteosarcoma is still controversial, which limits its clinical application. According to a randomized phase III trial, combining ZOL with chemotherapy has failed to improve the event-free survival, percentage of good histological response, and overall survival in patients with osteosarcoma (Piperno-Neumann et al., 2016). In addition, different studies have shown different effects of ZOL on inhibiting lung metastasis at the same dose. With the injection of 0.1 mg/kg ZOL twice a week, Wolfe et al. found no effect on metastasis (Wolfe et al. (2011)), while Labrinidis et al. observed tumor metastasis (Labrinidis et al. (2009)). Here, an HA-PEG-nHA NP loading ZOL system was constructed that not only verified the therapeutic effect of ZOL on osteosarcoma but also improved the drug utilization and the therapeutic effect both *in vitro* and *in vivo*.

Compared to the systemic administration of ZOL, the HA-PEG-nHA-ZOL NP delivery system possesses the following advantages to treat osteosarcoma. First, the NPs could realize the controlled release of ZOL, which can prolong the function time of drugs and reduce high dosage-induced side effects. HA acts as a ligand targeting osteosarcoma cells through CD44, and the binding of HA-CD44 and acid-sensitive detachment of PEG can realize specific targeting and drug release on osteosarcoma cells. In hybrid systems, PEG also helps increase the encapsulation rate of ZOL, improving the drug uptake and its deposit in tumors (Giger et al., 2013). Second, after complete drug release, the blank NPs could inhibit tumor cells as well, which has been proven in our previous research (Xu et al., 2021). Third, as an inorganic nucleus, nHA can repair tumor-induced osteolysis with good biocompatibility during bone repair (Dai et al., 2015). Additionally, previous studies have attempted the ZOL liposome encapsulation, but the drug delivery rate was less than 10% (Shmeeda et al., 2013). The loading rate of ZOL-supported polymer NPs was also low, for example, the ZOL loading rate of polylactic acid–hydroxy acetic acid NPs was only 1.4% (Li et al., 2019). Here, the drug delivery rate of NPs reached nearly 40% mainly because the oxygen atom of the phosphate in ZOL can chelate the calcium ion of nHA (Russell et al., 2008). The special P-C-P structure of bisphosphonates is called the bone hook. The high affinity of the bone hook for calcium promotes the bone-targeting effect of bisphosphonates, which has been widely used in clinical practice. For example, the radioisotope-labeled bisphosphonates (99mTc-HDP) can be used for bone imaging. Thus, the nano-drug delivery system could play an active role in bone repair after bone tumor resection.

The antitumor effect of HA-PEG-nHA-ZOL NPs has been shown in *in vitro* and *in vivo* experiments. First, using 143b, HOS, and MG63 osteosarcoma cells, we have validated the different effects between the free drug treatment and NP treatment *in vitro*. The cytotoxicity of the free drug application was much higher than that of the NPs. The NPs triggered osteosarcoma cell apoptosis by blocking the tumor cells in the S stage and inhibiting the proliferation of osteosarcoma cells. The antiapoptotic protein Bcl-2, mainly located in the outer membrane of the mitochondria, was upregulated in osteosarcoma to inhibit the apoptosis induced by various stimuli, injury, and cell survival signals (Valentin et al., 2018). Bax is an important pro-apoptotic gene (Lin et al., 2020). When overexpressed, Bax can bind to homologous dimers and promote apoptosis. The antiapoptotic protein Bcl-2 and pro-apoptotic protein Bax jointly regulate the expression of the pro-apoptotic factor caspase 3 (Ma et al., 2020). HA-PEG-nHA-ZOL NP-induced upregulation of BAX, Bcl-2, and caspase 3 destroyed the tumor metabolism balance and triggered the tumor apoptosis. Here, the NP was designed to specifically kill tumor cells and avoid the side effects of excessive ZOL use, as mentioned before. Therefore, NPs and saline were injected into orthotopic osteosarcoma rat models, respectively. NPs produced positive therapeutic effects on osteoblastoma with few systemic side effects.

Since ZOL possesses significant therapeutic effects on osteosarcoma, methods to improve its stability and pharmacokinetic properties to enhance its efficacy are needed. Nanomedicine has increasingly been used in cancer diagnosis and treatment because it can modulate the biological distribution and target abundant chemotherapy drugs, thereby reducing drug toxicity (Garbayo et al., 2020). Drug delivery systems protect drugs from degradation in the biological environment and deliver them in a controlled manner (Nikezić et al., 2020). A delivery system using ZOL and calcium loaded with doxorubicin as the nanocore and VEGF-modified erythrocyte membranes as the nano-shell (V-RZCD) has been constructed (Wu et al., 2021b). In addition to the antitumor effects of the chemotherapy drug, the framework can also monitor drug aggregation at the tumor site based on doxorubicin’s red fluorescence. The V-RZCD targets osteosarcoma cells and escapes immune recognition under the disguise of the erythrocyte membrane. Moreover, the acidic tumor microenvironment provides an opportunity to develop pH-responsive drug delivery systems (Liu et al., 2014). A calcium carbonate core-cross-linked NP of methoxy poly(ethylene glycol)-block-poly(l-glutamic acid) through mineralization for intracellular delivery of doxorubicin has been developed (Li et al., 2020). This system has high drug loading capacity and pH-dependent drug release. Moreover, the drug loading rate and releasing time of NPs could be prolonged if they are encapsulated in hydrogel, which would be better for topical delivery. In this study, we intended to research whether the NPs could induce tumor cell apoptosis and necrosis directly. To achieve the goal of antitumor and bone repairing simultaneously, we plan to encapsulate the NPs in hydrogel for topical delivery in the future study.

Since the purpose of this study was to preliminarily test the inhibitory effect of the NPs on bone tumors *in vivo*, a local intratumor injection method was performed. This method possessed a relatively simple operation, but there may exist some distance from the actual situation. Following this, the establishment of a bone tumor resection model combined with NPs may be promising to intuitively detect the effect of NPs on bone tumor recurrence. In addition, the mechanism of tumor inhibition by NPs *in vivo* has not been verified, which will be further explored in subsequent studies. Taken together, the nano-drug delivery system possesses great potential for future cancer clinical application.

## 5 Conclusion

The HA-PEG-nHA-ZOL nanoparticles can effectively inhibit the proliferation of a series of osteosarcoma cell lines, with low cell cytotoxicity on normal cells. This effect is achieved by the specific binding of PEG to CD44 receptors on the tumor cell surface, through which it enhances the tumor cell apoptosis rate and apoptosis-related protein expression. *In vivo* experiments demonstrated that the local injection of HA-PEG-nHA-ZOL NPs stimulated tumor necrosis and apoptosis, as well as granulocyte infiltration in blood vessels. This endows the ZOL nano-drug delivery system with great potential for local treatments to prevent local tumor recurrence in clinical therapy.

## Data Availability

The original contributions presented in the study are included in the article/Supplementary Material, further inquiries can be directed to the corresponding author.
