# Corrigendum: Therapeutic effects of zoledronic acid-loaded hyaluronic acid/polyethylene glycol/nano-hydroxyapatite nanoparticles on osteosarcoma

**DOI:** 10.3389/fbioe.2025.1601751

**Published:** 2025-05-07

**Authors:** Yan Xu, Jingqi Qi, Wei Sun, Wu Zhong, Hongwei Wu

**Affiliations:** ^1^ Hunan Cancer Hospital and The Affiliated Cancer Hospital of Xiangya School of Medicine, Central South University, Changsha, China; ^2^ Zhejiang University-University of Edinburgh Institute, Haining, China

**Keywords:** nanoparticle, osteosarcoma, zoledronic acid, tumor therapy, targeted therapy

In the published article, there was an error in Figure 2 as published. In Figures 2C, D, an image was mistakenly duplicated. The corrected Figure 2 and its caption appear below.

**FIGURE 2:**
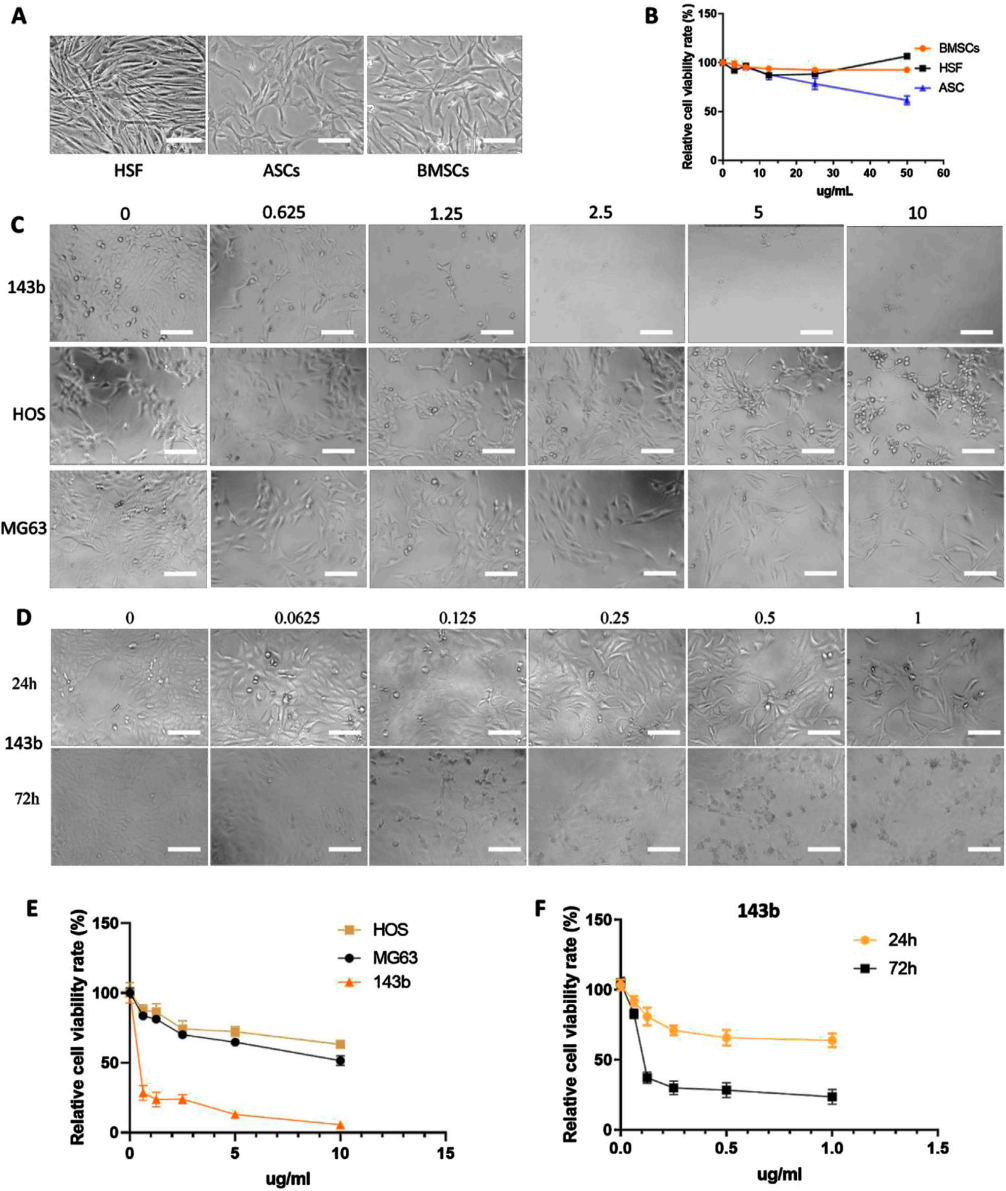
Effect of nanoparticles on three types of normal cells and three osteosarcoma cell lines. **(A)** Photoscopic images of three normal cell types (HSFs, ASCs, and BMSCs) treated with 0–50 μg/mL concentrations of nanoparticles. **(B)** Relative cell viability of three normal cell lines under 0–50 μg/mL concentrations of nanoparticles. **(C, E)** Photoscopic images and relative cell viability of three kinds of osteosarcoma cells (143b, HOS, and MG63) treated with 0–10 μg/mL concentrations of nanoparticles. **(D, F)** Photoscopic images and relative cell viability of 143b cells treated with 0–1 μg/mL concentrations of nanoparticles. Scale bar = 100 um.”

The authors apologize for this error and state that this does not change the scientific conclusions of the article in any way. The original article has been updated.

