# Supplementary material for: Therapeutic Effects of Zoledronic Acid-Loaded Hyaluronic Acid/Polyethylene Glycol/Nano-Hydroxyapatite Nanoparticles on Osteosarcoma
Source: Front Bioeng Biotechnol. 2022 May 26;10:897641. doi: 10.3389/fbioe.2022.897641 (PMC9181619; doi:10.3389/fbioe.2022.897641)
Supplement: Supplementary file 1 [file DataSheet1.PDF]

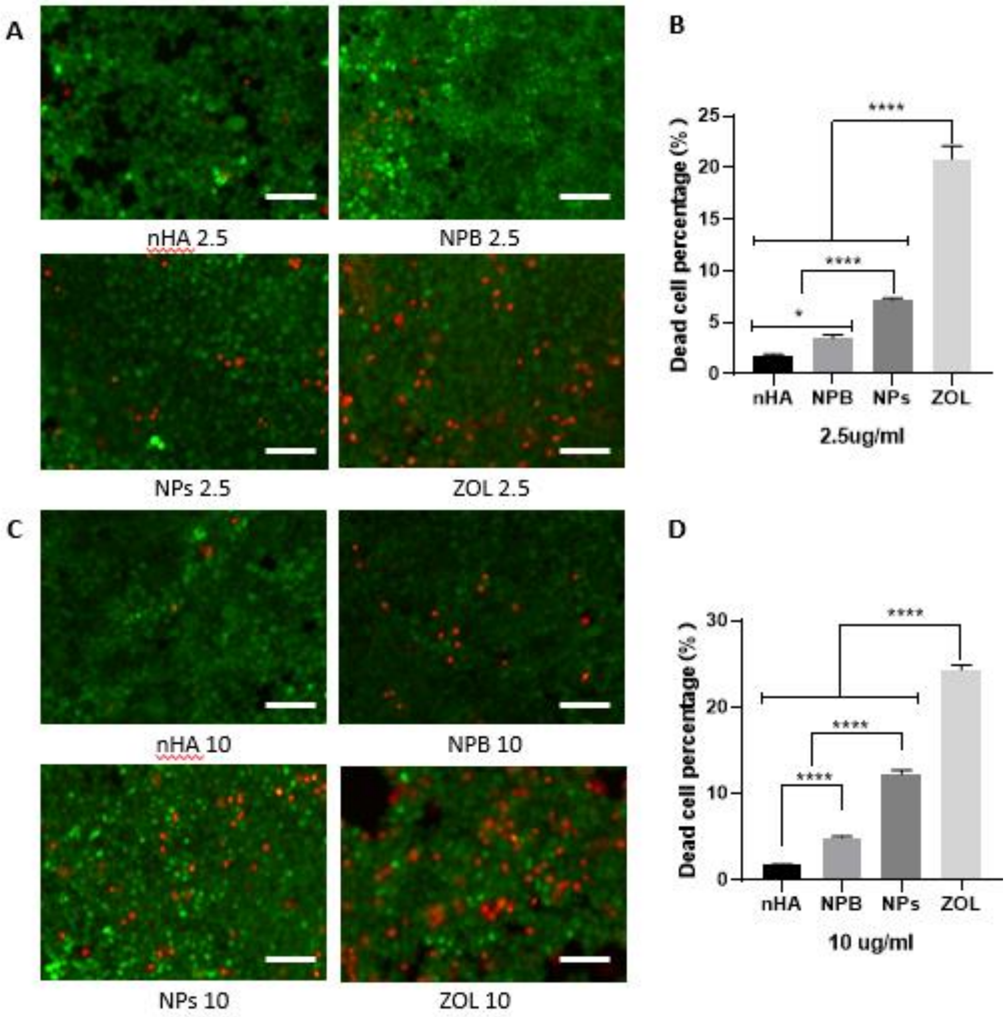

Figure S1 Live/dead staining and comparison of 143b cells treated with nHA, NPB, NPs and ZOL, respectively. A and B: the concentration of the nanoparticles or drug was 2.5 ug/ml. C and D: the concentration of the nanoparticles or drug was 10 ug/ml.

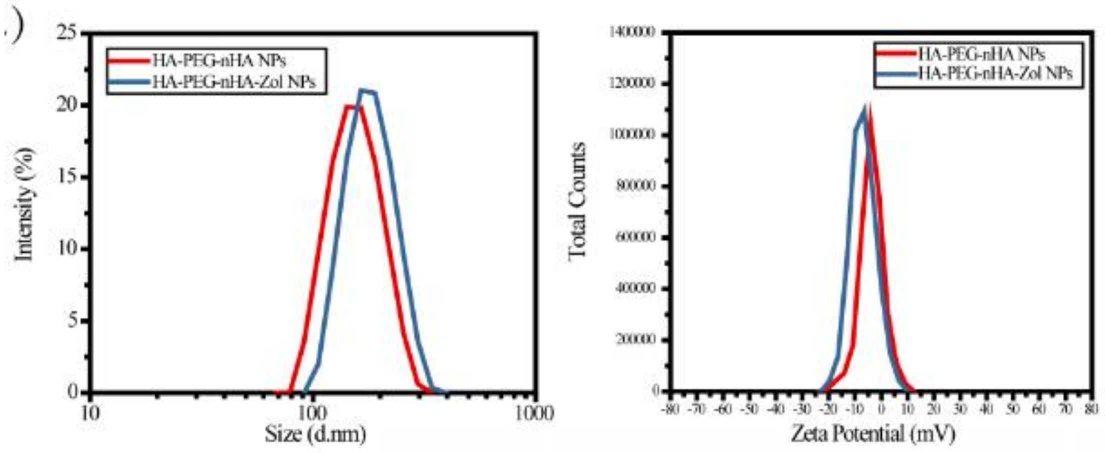

Figure S2 Size distribution and Zeta potential of the nanoparticles.

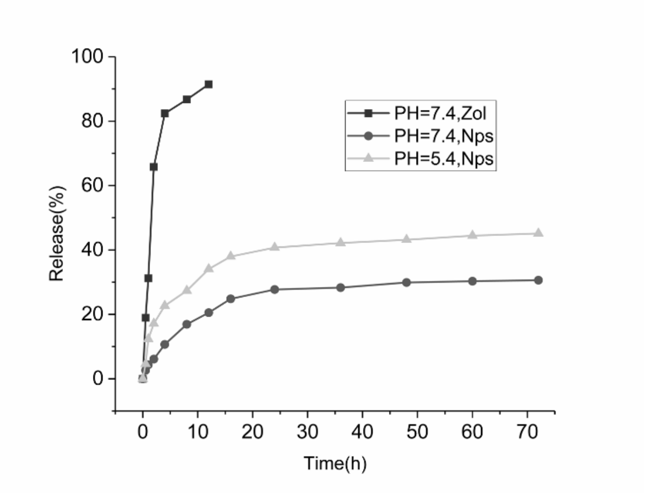

Figure S3 drug release curve of the nanoparticles.

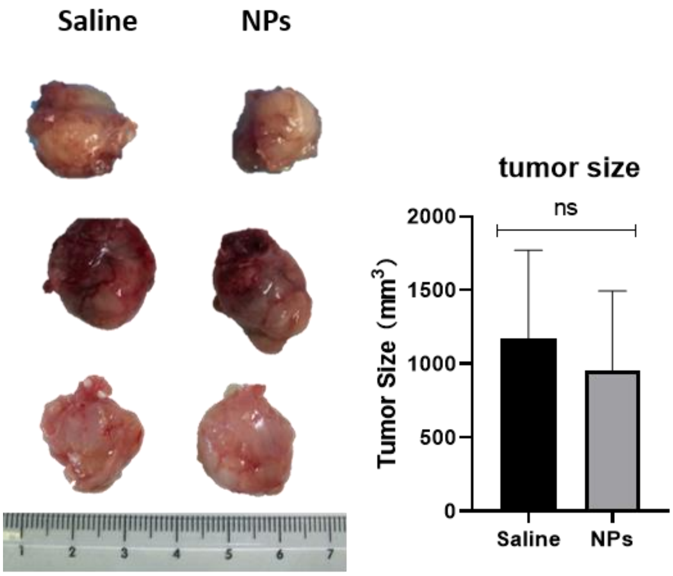

Figure S4 Tumor size comparison between saline and nanoparticle treated group.

Table 2. Size, Zeta Potential and PDI of NPs

Name of NPs	Size (d.nm)	Zeta Potential (mV)
HA-PEG-nHA NPs	159.0±2.3	6.01±0.23 mV
HA-PEG-nHA-Zol NPs	182.5±1.7	7.03±0.19 mV
